# De novo male breast metastases from nasopharyngeal carcinoma: a case report

**DOI:** 10.1186/s13256-024-04353-1

**Published:** 2024-01-30

**Authors:** Saida Sakhri, Ines Zemni, Mohamed Ali Ayadi, Fethia Abidi, Yoldez Houcine, Sonia Sghaier, Tarek Ben Dhiab

**Affiliations:** 1 Department of Surgical Oncology, Salah Azaiez Institute, Faculty of Medicine of Tunis, University Tunis EL Manar, Tunis, Tunisia; 2 Laboratory Microorganisms and Active Biomolecules, Sciences Faculty of Tunis, University Tunis El Manar, Tunis, Tunisia; 3 Department of Radiology, Salah Azaiez Institute, Faculty of Medicine of Tunis, University Tunis EL Manar, Tunis, Tunisia; 4 Depatment of Pathology, Salah Azaiez Institute, Faculty of Medicine of Tunis, University Tunis EL Manar, Tunis, Tunisia; 5 Department of Radiology, Military Hospital of Instruction of Tunis, Faculty of Medicine of Tunis, University Tunis EL Manar, Tunis, Tunisia

**Keywords:** Nasopharynx, Male, Breast, Synchronous metastasis, Undifferentiated carcinoma

## Abstract

**Background:**

Nasopharyngeal carcinoma is known for its high potential for regional and distant metastasis. However, breast metastasis is rarely reported.

**Case presentation:**

A 39-year-old Caucasian male presented with bilateral neck lymph node enlargement. Radiological examination with contrast-enhanced computed tomography scan and breast imaging revealed an enhancing mass lesion in the right breast. Histopathology of the nasopharynx mass was suggestive of undifferentiated nasopharyngeal carcinoma. A breast biopsy confirmed the diagnosis of synchronous breast metastasis from the nasopharyngeal carcinoma. We present this study to illustrate that Nasopharyngeal carcinoma can metastasize to the male breast. Furthermore, the high incidence of nasopharyngeal carcinoma metastasis underscores the pressing need to identify effective and safe strategies, emphasizing the importance of utilizing computed tomography scans for metastasis detection.

**Conclusion:**

The present study illustrates the first case of synchronous male breast metastases from nasopharyngeal carcinoma. Thus, it is critical to distinguish between metastatic pathology and coexisting second malignancies to plan appropriate therapy.

## Introduction

Nasopharyngeal carcinoma (NPC) is a rare malignancy with a frequency of 0.5–2 per 100,000 in Europe and the USA [1]. It primarily affects young and middle-aged adults. The incidence is higher in the Chinese and Tunisian populations; these areas are considered endemic for NPC [1]. It is associated with the highest rate of cervical lymph node and distant metastasis, which usually involve the bones and the lungs [2, 3].

However, the mammary gland is an infrequent site of metastasis. It accounts for approximately 2% of all mammary malignancies and would be incredibly rare if it happened in a male patient, such as in the present case. To our knowledge, there have only been a few rare cases of metastatic nasopharyngeal carcinoma to the mammary gland reported in literature [4].

## Case presentation

In March 2021, a 39-year-old  Caucasian man with no medical, family, and psychosocial history presented with a lump in the right upper neck region that had been evolving for about 4 months, associated with a 2-month history of headache and epistaxis. Personal medical and surgical history was unremarkable. Physical examination revealed multiple bilateral neck node enlargements, with the largest measuring 10 cm × 1.5 cm in the right cervical region. Fiberoptic nasopharyngoscopy revealed lifting of the right side of the tonsillar crypts, and the surface was rough. This lesion was biopsied.

Histopathology findings of the nasopharynx specimen showed characterized large malignant cells with vesicular nuclei, prominent nucleoli, syncytial growth pattern, and a lymphoplasmacytic infiltrate. These findings are consistent with undifferentiated NPC (World Health Organization type III).

**Fig. 1:**
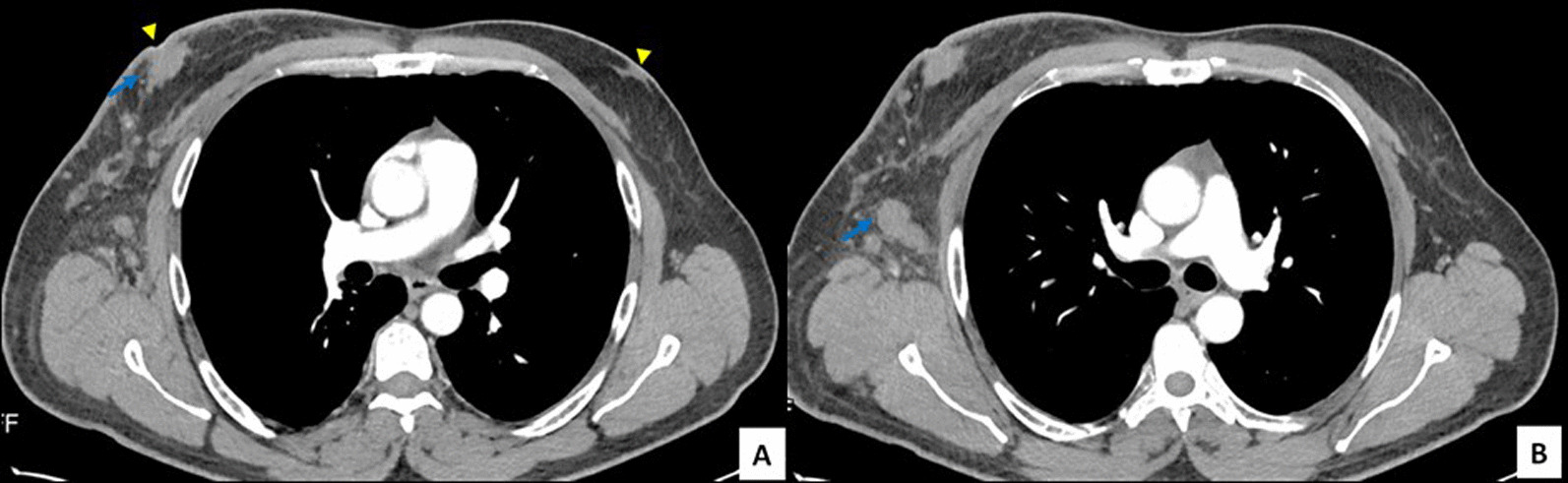
Arterial phase thorax CT (mediastinal window axial cuts). ** A** demonstrates bilateral gynecomastia (arrowheads) with enhancement right retroareolar mass (blue arrow) and **B** ipsilateral lymph node enlargement (blue arrow)

**Fig. 2:**
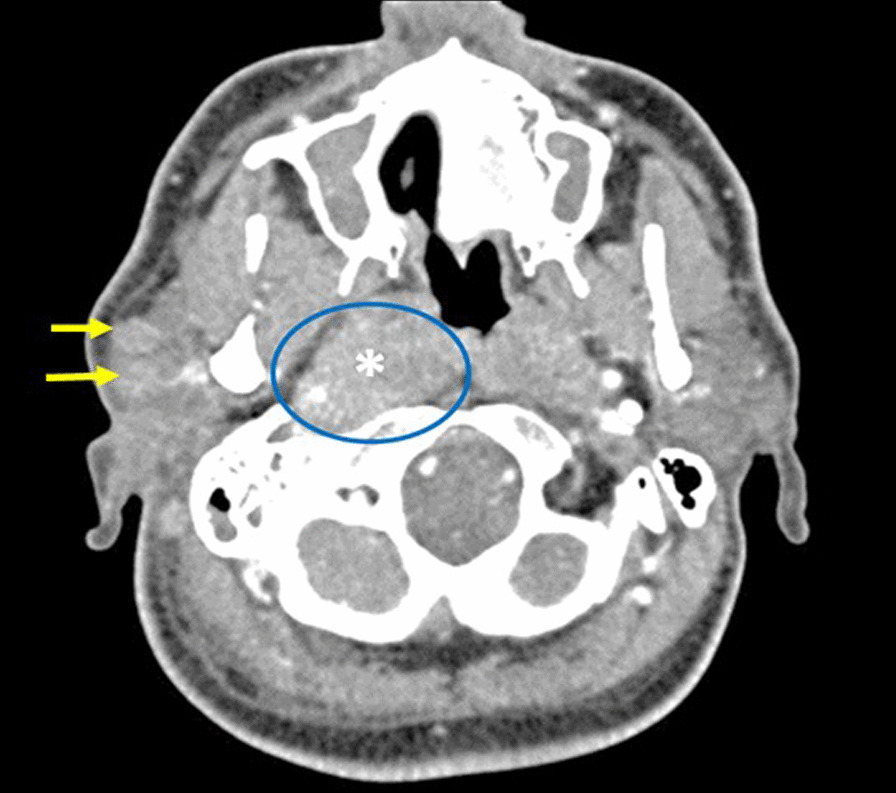
Venous phase cervical CT (soft tissue window axial cut) shows nasopharyngeal carcinoma centered in right Rosenmüller fossa (asterisk) with deep posterior extension into carotid space and heterogeneous masses (arrows) of the right parotid

Radiological examination with contrast-enhanced computed tomography (CECT) scan of the head and the neck showed a nasopharyngeal lump centered in the right Rosenmüller fossa with deep extension into carotid space (Fig. 1) and bilateral cervical lymphadenopathy with a maximum node size of almost 9 cm × 1.5 cm. CECT of the chest revealed a heterogeneously enhancing lesion in the right breast parenchyma (in the retroareolar area) with ipsilateral lymph node enlargement (Fig. 2).

The breast examination found no apparent anomaly, and there were no axillary palpable nodes. No other abnormalities were seen in the rest of the somatic examination. The results of routine blood examination were within the normal range.

**Fig. 3:**
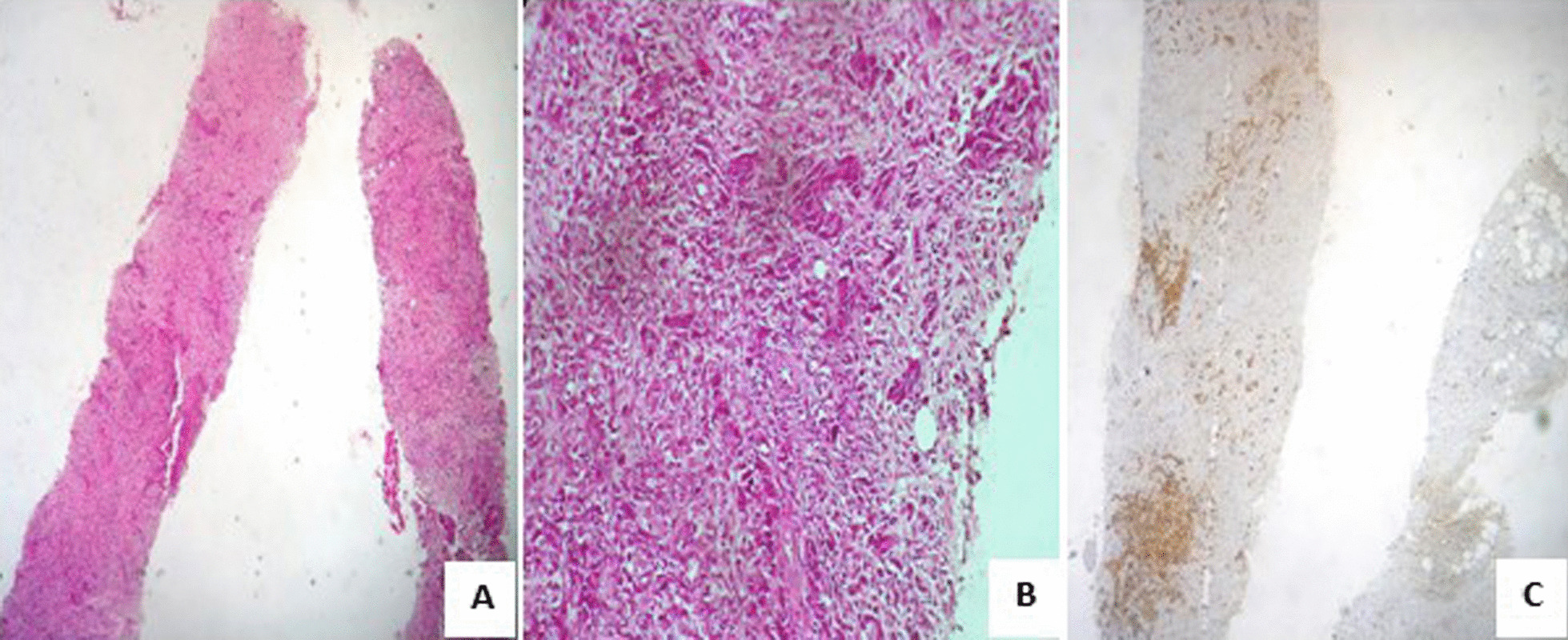
Breast biopsy histological study with immunohistochemistry. ** A** biopsy cores entirely tumoral dissociated by an undifferentiated malignant epithelial proliferation (HE × 5). **B** large tumor cells with atypical nuclei (HE × 10). **C** tumor cells expressing p40 (HE × 20)

The patient then underwent a breast ultrasound that showed two oval microlobulated hypoechoic masses without speculations measuring 10 mm and 12 mm, respectively, located in the upper outer quadrant and the retroareolar region of the right breast, associated with the ipsilateral axillary lymph node. Our patient underwent an ultrasound-guided biopsy; histologically, the cores examined were dissociated by undifferentiated carcinomatous proliferation. The tumor cells were large with reduced cytoplasm. They had atypical and often mitotic nuclei. In immunohistochemistry, they expressed p40 and p63 (Fig. 3). They were negative for GATA 3, mammaglobin, and hormone receptors. These data confirmed the diagnosis of breast metastasis from the NPC. The patient was thus diagnosed to have NPC stage T2N3M1.

After a multidisciplinary meeting, palliative treatment was indicated. The patient was treated with several lines of chemotherapy and a palliative external beam radiation therapy on the nasopharynx and cervical lymph nodes; he received 40 Gy in 20 fractions in view of the stability of the disease to relieve symptoms and reduce primary tumor burden. Breast radiation has been planned for this patient, but he died after 30 months of follow-up following a deterioration of the general and respiratory state.

## Discussion

Metastasis to the breast from extramammary carcinomas is extremely rare; the incidence is reported to be only 0.5–2% [2, 5]. The first case of extramammary breast metastasis was reported in 1903. Lymphomas and melanomas are the most prevalent causes of extramammary metastases to the breast [6]. However, the breast is an extremely unusual site of metastatic NPC. It typically reflects a disseminated disease that causes breast metastasis [3, 7].

NPCs develop distant metastasis in about 30% of cases; it can be found at their initial diagnosis or after diagnosis [8]. Most metastases (90%) occur within 3 years after the end of radiotherapy. In the present case, breast metastasis was found in the initial extension assessment because the mass was not identified in the physical examination. Usually, breast metastasis occurs at an advanced stage as the result of disseminated cancer [9, 10]. In literature, the majority of cases were diagnosed months after the end of NPC treatment, unlike our case. Therefore, those patients were considered to be in the palliative stage, and they received only chemotherapy; none of the patients underwent breast surgery [2, 4, 5, 10], However, in the present case, the diagnosis was made during the initial CT scan. As a result, our patient underwent radiotherapy and chemotherapy.

Usually, NPC involves metastasis to regional lymph nodes, but the mechanism by which nasopharyngeal carcinoma metastasizes to the mammary gland is not well identified. It may be a result of axillary lymph node metastasis through retrograde lymphatic drainage. NPC is adjacent to a rich lymphatic system, so it often metastasizes to cervical lymph node levels I, II–IV, and levels II and IV lymph nodes [11]. However, NPC can also lead to axillary lymph node invasion, attributed to retrograde lymphatic drainage. Axillary lymph node drainage occurs due to the obstruction of the cervical canal. This is observed in patients whose lymphatic drainage channels have been previously significantly altered, either through lymphadenectomy, radiotherapy, or tumor recurrence [2, 5]. The extensive cervical lymph node invasion in our patient may explain the retrograde invasion of the axillary lymph nodes.

Breast metastases may be symptomatic (palpable mass) or diagnosed by radiology. They can often be misdiagnosed as primary breast cancer, due to their similarity to the primary breast cancer [4]. In the literature, only four cases presented with bilateral breast metastases [10], but most cases, including this one, presented with unilateral breast tumors [9]. Radiologically, distinctive architectural distortion, microcalcification, and speculation linked to primary breast malignancy are uncommon in metastatic lesions [2, 5]. CT scan can be helpful for diagnosis, revealing breast lesions, especially in the case of male patients where mammography is not systematically indicated. Despite the limited sensitivity and specificity of breast lesion detection, it can also identify axillary metastasis. Therefore, it should be utilized as an additional assessment tool [12]. Only nasopharyngeal and mammary biopsy and histological examination can confirm the diagnosis [4]. Immunohistological examination of the tumor tissue is crucial for diagnosis and to rule out primary breast cancer. In fact, it revealed positivity for pan cytokeratin (PCK) and CK17, while testing negative for CK20, CD3, CD20, chromogranin A, and synaptophysin. However, primitive breast tissue showed negativity for CD3, CD20, chromogranin A, and synaptophysin [2]. So that we can confirm the diagnosis of breast metastasis.

In all previously reported cases in the literature, breast metastasis following the end of treatment generally occurs at an advanced stage. The median time to breast metastasis ranged from 3 to 39 months [4]. In our case, the metastasis was diagnosed concurrently with the primary NPC. Consequently, treatment remains a real challenge, given the absence of a standard therapy for synchronous metastasis. The decision to opt for palliative or curative treatment must be carefully deliberated by the medical committee. However, in case of recurrent or distant metastasis that occurs after loco regional therapy, systemic chemotherapy based on platinum is considered the preferential first-line treatment, and as a second-line regimen, the docetaxel, cisplatin, 5-FU, and leucovorin combined with radiotherapy are used to control the distant metastasis [3, 10]. In the case of breast metastasis, some authors recommended combining systemic chemotherapy and loco regional radiotherapy; however, mastectomy may not have a benefit on overall survival (OS), so it is important to avoid the surgery and to use systemic therapy. Similarly to our patient, which diagnosis of breast metastasis and NPC was synchronous, he initially received systemic chemotherapy.

Despite systemic therapy, the prognosis is poor, and the median survival time following diagnosis ranges between 82 and 190 months. Tian subdivided patients into two groups of metastatic disease, which were M1a (single organ or 1–5 lesions) and M1b (multiple organ or > 5 lesions). The 5‐year OS rates for M1a disease and M1b disease were 38.7% and 7.0%, respectively (*p* < 0.01) [13]. In addition, It is demonstrated that the leading cause of mortality in patients with nasopharyngeal carcinoma is distant metastasis [2, 9].

In nasopharyngeal cancer with de novo metastases, sequential locoregional treatment with variable doses of chemotherapy and radiotherapy has demonstrated its efficacy in retrospective studies [14, 15] and recently in a prospective randomized study by Rui You *et al*. using high doses of radiotherapy, with a positive impact in terms of PFS and OS [16].

Our case is interesting but has certain limitations; the IHC was not well detailed, and the Epstein–Barr virus test was not carried out, despite its value in detecting recurrence and metastasis.

## Conclusion

This case highlights the importance of clinical correlation with biopsy specimens due to the similarity of the two cancers. The radiographic and histological features of breast metastasis from NPC closely resemble those of primary breast carcinoma. Clinical presentation is not specific for metastases, due to the rarity of the breast localization. Immunohistochemistry is highly valuable in such cases, and confirming the diagnosis is aided by the Epstein–Barr virus test. It is therefore important to make a clear distinction between metastatic and primary breast lesions. This is a crucial step that influences and impacts therapeutic strategy and helps avoid unnecessary surgery. A multidisciplinary approach is therefore always beneficial in making the diagnosis and managing the patient appropriately.

## Data Availability

Data supporting our findings were taken from the patient’s folder.

## Abbreviations

NPC: Nasopharyngeal carcinoma
CECT: Contrast-enhanced computed tomography
